# Effects of Adenotonsillectomy on Neurocognitive Function in Pediatric Obstructive Sleep Apnea Syndrome

**DOI:** 10.1155/2014/520215

**Published:** 2014-05-26

**Authors:** Fumie Horiuchi, Yasunori Oka, Kenjiro Komori, Yasumasa Tokui, Teruhisa Matsumoto, Kentaro Kawabe, Shu-ichi Ueno

**Affiliations:** ^1^Department of Neuropsychiatry and Neuroscience, Ehime University Graduate School of Medicine, Shitsukawa, Toon-city, Ehime 791-0295, Japan; ^2^Center for Sleep Medicine, Ehime University Hospital, Ehime, Japan; ^3^Hiroshima Sleep Center, Hiroshima, Japan; ^4^Zaidan Niihama Hospital, Niihama, Ehime, Japan

## Abstract

Obstructive sleep apnea syndrome (OSAS) in children does not only present with symptoms of sleep disturbances but also with associated symptoms such as growth failure, enuresis, academic learning difficulties, and behavioral problems, including attention deficit/hyperactivity disorder- (ADHD-) like symptoms. We evaluated neurocognitive functions before and after adenotonsillectomy in a patient with OSAS. An 11-year-old boy suspected of having ADHD with nocturnal enuresis was referred for evaluation. He was found to have adenotonsillar hypertrophy. Presence of snoring was evident only after detailed medical interview. Polysomnography confirmed the diagnosis of OSAS, which was subsequently treated by adenotonsillectomy. The apnea/hypopnea index decreased from 21.9 at baseline to 1.8 after surgery, and the frequency of enuresis fell from almost nightly to 2-3 times per month. Neurocognitive and behavioral assessment after the treatment of OSAS showed significant improvement in cognitive functions, especially attention capacity and considerable amelioration of behavioral problems including ADHD-like symptoms. As the most common cause of pediatric OSAS is adenotonsillar hypertrophy, medical interview and oropharyngeal examination should always be performed in children suspected of having ADHD. The necessity of sleep evaluation for children with ADHD-like symptoms was also emphasized.

## 1. Introduction

Obstructive sleep apnea syndrome (OSAS) is a common disorder in adults as well as in children. The prevalence of pediatric OSAS is up to 5.7% [1–3]. The clinical symptoms of pediatric OSAS are not always compatible with those of adult OSAS. A wide range of symptoms and signs are associated with pediatric OSAS in the developmental stages. Diagnosis of OSA in children is made on the basis of sleep history, physical examination, and polysomnographic findings. In the diagnostic criteria of pediatric OSAS by the International Classification Sleep Disorder, second edition (ICSD-II), witnessed snoring and/or labored/obstructed breathing during the child's sleep is required. In addition, the caregiver reporting at least one of the following is necessary: paradoxical inward rib-cage motion during inspiration, movement arousals, diaphoresis, neck hypertension during sleep, excessive daytime sleepiness/hyperactivity, aggressive behavior, morning headaches, and secondary enuresis [4]. Untreated pediatric OSAS has been related with various problems such as attention deficit/hyperactivity disorder (ADHD), poor academic achievement, and behavioral problems [5]. The most common cause of pediatric OSAS is adenotonsillar hypertrophy and the peak age of pediatric OSAS is 3–7 years, which parallels the age of adenotonsillar enlargement [6].

ADHD is a neuropsychiatric syndrome affecting 3–16% of school children with world prevalence of 5.29% [7]. The diagnosis of ADHD is sometimes difficult because other psychiatric and medical disorders such as anxiety disorder and sleep disorder can also mimic ADHD symptomatology [8].

There is a well-established correlation between sleep disturbances and ADHD [9]. In the case of pediatric OSAS due to adenotonsillar hypertrophy, the surgery may make it possible to improve behavioral and cognitive performance, although parents and even physicians do not necessarily associate these behavioral and neurocognitive deficits with OSAS.

The aim of this study was to investigate the necessity of identifying pediatric OSAS for children with ADHD symptomatology and to assess the effects of treatment of OSAS on neurocognitive functions.

## 2. Case Presentation

An 11-year-old boy was referred to the university hospital with suspected ADHD. He tended to be hyperactive since he started crawling, and he has been still fidgeting at school, with academic problems related to inability to concentrate on work. The socioeconomical level of his family was on average. ADHD was suspected by teachers based on forgetfulness of school needs and homework. His symptoms were compatible with the diagnostic criteria of ADHD, predominantly inattentive type, based on the diagnostic criteria of Diagnostic and Statistical Manual, fourth edition, text revision (DSM-IV-TR) [10]. The medical history also included nocturnal enuresis almost every night, treated with oxybutynin and desmopressin, though both medications were ineffective.

Physical examination showed height within the normal range (155 cm), body weight of 48.4 kg, and normal body mass index (20.0 kg/m^2^, Rohrer index: 129.2). His parents had not noticed his snoring until they had the detailed medical interview. Oropharyngeal examination showed adenotonsillar hypertrophy with Mackenzie stage II to stage III. Presence of snoring was evident only after medical interview to the parents. Based on suspicion of OSAS, the patient was scheduled for attended polysomnography (PSG) with video monitoring and multiple sleep latency test (MSLT). PSG recording included electroencephalography (EEG) (C3-A2 and C4-A1), electrooculography, submental and bilateral lower limb electromyography, and electrocardiography, together with continuous recording of oronasal airflow, chest and abdominal movements, and oxygen saturation. Sleep stages were scored according to the Rechtschaffen and Kales manual [11]. Apneas were scored when the drop of flow by ≥90% of preevent baseline for at least the duration of 2 breaths. Hypopneas were scored when the signal dropped by ≥30% for at least the duration of 2 breaths with ≥3% oxygen desaturation. All-night PSG showed frequent episodes of obstructive sleep apnea/hypopnea (apnea/hypopnea index = 21.9/hour) associated with falls in oxygen saturation and EEG arousals.

He also underwent a series of neurocognitive tests and his parents filled out questionnaires. The cognitive test battery consisted of the Japanese version of the Wechsler Intelligence Scale for Children-Third Edition (WISC-III), Kaufman Assessment Battery for Children (K-ABC), for assessment of intelligence and achievement, the Rey-Osterrieth Complex Figure Test (ROCF) as a scale of visuospatial construction, and the Rey's Auditory Verbal Learning Test (RAVLT) to assess verbal learning and memory [12–15]. For behavioral assessment, the parents' version of the Child Behavior Checklist (CBCL) was used to measure social competency and problematic behavior [16].

Based on the diagnosis of pediatric OSAS, he underwent adenotonsillectomy.

He was reevaluated for PSG, MSLT, and neurocognitive and neurobehavioral assessment 4 months after surgery. After adenotonsillectomy, the frequency of enuresis reduced to 2-3/month and his snoring disappeared. Although he did not complain about sleepiness before the operation, he reported improvement in daytime sleepiness and concentration after adenotonsillectomy.

**Figure 1:**
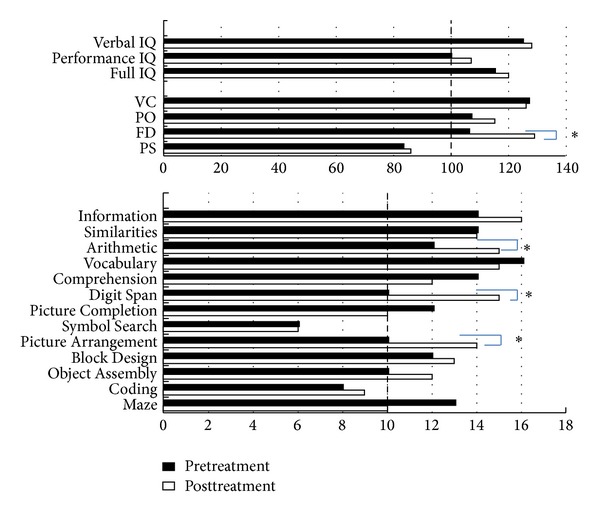
Wechsler Intelligence Scale for Children-Third Edition (WISC-III) scores of IQ, 4 factors and 13 subscores before and after treatment. Verbal IQ is calculated by the scores of  “Information,” “Similarities,” “Arithmetic,” “Vocabulary,” and “Comprehension.” Performance IQ is calculated by the scores of “Picture,” “Comprehension,” “Coding,” “Picture,” “Arrangement,” “Block Design,” and “Object Assembly.” Full IQ = Verbal IQ + Performance IQ. VC (verbal comprehension) is calculated by the scores of “Information,” “Similarities,” “Vocabulary,” and “Comprehension.” PO (perceptual organization) is calculated by the scores of “Picture Comprehension,” “Picture Arrangement,” “Block Design,” and “Object Assembly.” FD (freedom of distraction) is calculated by the scores of “Arithmetic” and “Digit Span.” PS (performance speed) is calculated by the scores of “Coding” and “Symbol Search”. *P < 0.05.

**Figure 2:**
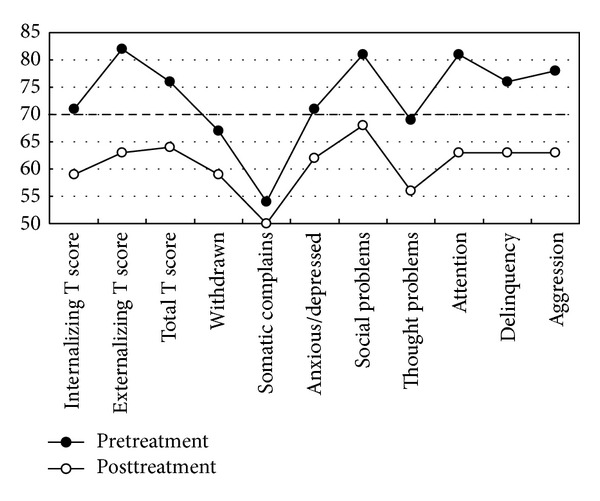
Child Behavior Checklist scores of general scores and subscores before and after treatment.

Table 1 lists the results of PSG and MSLT conducted before and after adenotonsillectomy. The apnea/hypopnea index decreased to 1.8 after treatment, and the mean sleep latency of MSLT increased from 14.2 minutes to 17.2 minutes. On the WISC-III, the score of FD increased significantly from 106 to 129, but no differences were noted in the scores of VIQ, PIQ, and FIQ. With regard to the 13 subtests, the scores of Arithmetic, Picture Arrangement, and Digit Span subtests showed significant improvement (Figure 1). Furthermore, the score of Simultaneous Processing Scale of the K-ABC increased from 95 (below the average score of 100) to 112 (Table 2). The immediate and delayed recall trial scores of the ROCF improved, and the recall trial scores of RAVLT also showed large increases (Table 3). In addition, all three general scales of the CBCL, Internalizing T score (from withdrawn behavior, somatic complains, and anxious/depressed), and Externalizing T score (from delinquency and aggression) improved from clinical range to almost normal range. Out of 8 subscale scores of the CBCL, social problems, attention, delinquency, and aggression were above clinical cutoff value (70 points) before treatment and all subscores became below the clinical cutoff values after adenotonsillectomy (Figure 2).

## 3. Discussion

The present study demonstrated that adenotonsillectomy for severe pediatric OSAS resulted in significant improvement in nocturnal ventilation, sleep architecture, snoring, neurocognitive functions, especially attention capacity, and behavioral problems, including ADHD-like symptoms.

ADHD in children is associated with various sleep disorders including pediatric OSAS, periodic limb movement (PLMD), enuresis, and delayed sleep phase syndrome (DSPS) [17–21]. According to a meta-analysis of both subjective and objective studies, the incidence of sleep disturbances was significantly higher among children with ADHD as compared to a control group [22]. Naseem et al. [23] suggested that sleep disorders, especially sleep apnea, might be one of the underlying causes of ADHD.

On the other hand, patients with sleep disturbances often display behavioral patterns that resemble some features of ADHD. Parents of children with OSAS tend to focus solely on daytime behavior problems, inattentiveness, and academic failure, rather than sleep problems. Therefore, these children are prone to be diagnosed with neurodevelopmental disorders, such as ADHD. Our patient was referred to the hospital with suspected ADHD, not OSAS, and initially fulfilled the diagnostic criteria of ADHD. It is often difficult to reveal which of the disorders is the primary and which is a byproduct of the other [9]. Moreover, in this case, parents were not aware of snoring before being pointed out by the physician. According to Chervin et al, snoring was strongly predictive of a future diagnosis of hyperactivity over the long-term [24], while children with primary snoring had a higher risk for hyperactive and inattentive behavior, compared to children who never snored [25]. Sleep is not always assessed in children suspected of having ADHD, but children with ADHD should be assessed for quality of sleep, checked for enlarged tonsils, and interviewed for presence of snoring. Sleep studies should be taken into consideration in children suspected of having OSAS as an underlying cause of ADHD-like symptoms. The use of stimulants is the most common therapeutic trial for ADHD. However, treatment of the underlying sleep disorder can avoid unnecessary use of potent and sometimes harmful medications [26].

The most common cause of nocturnal enuresis is neurological-developmental delay, but pediatric OSAS is occasionally associated with enuresis [27]. Brouilette et al. [28] reported a higher incidence of enuresis in children with OSAS compared with the control group. They also reported that 35% of children with adenohypertrophy suffered from enuresis and that adenotonsillectomy significantly improved enuresis. The high prevalence of enuresis in children with OSAS is thought to be due to the effects of obstructive sleep apnea on arousal response, bladder pressure, and urinary hormone secretion [29]. In addition, enuresis is also common in children with ADHD, although the mechanism of enuresis in ADHD remains elusive. Previous studies have demonstrated a significantly increased prevalence of ADHD in children with enuresis [30]. Fifteen percent of all enuretic children were diagnosed with the full syndrome of ADHD, and 22.5% met the criteria of the ADHD inattentive subtype [31]. Children with ADHD and drug resistant enuresis should be assessed for sleep in consideration of the possibility of OSAS.

The performance on the K-ABC test can reflect the ability of a child for academic achievements [32]. In our case, adenotonsillectomy resulted in marked improvement in the Simultaneous Processing Scale. This change enabled the child to reach the original abilities and fulfilled the cognitive potential. The effects of adenotonsillectomy emphasize OSAS as the cause of underachievement [33]. Adenotonsillectomy also significantly improved the Arithmetic, Picture Arrangement, and Digit Span subtests of the WISC-III. These cognitive functions are related to attention capacity and were probably related to the academic problems before the treatment. Based on previous reports [34–37], the most frequently reported neurocognitive impairment in patients with OSAS is verbal short-term memory, attention capacity, and problem-solving strategies. In our case, FIQ, VIQ, and PIQ were not significantly different before and after adenotonsillectomy, suggesting that OSAS did not affect IQ itself. However, the ROCF and RAVLT scores improved after treatment, and such improvement could be related indirectly to the improvement in short-term memory and attention capacity. Moreover, the ROCF reflects not only visuoperceptual and visuoconstructional ability, but also executive function, especially planning and organization [38]. The executive function could also improve after treatment. Friedman et al. [33] reported improvement in the mean scores of various cognitive tests in patients with OSAS after adenotonsillectomy, to levels similar to those of the healthy controls. They concluded that the neurocognitive deficit was mostly reversible. However, at which point the impaired neurocognitive function becomes irreversible is not clear at this stage. Undoubtedly, disturbances of neurocognitive function have a significant impact on the children's physical and mental development. Further research should address the reversibility of various neurocognitive functions in OSAS.

With regard to the behavior problems, externalizing behavior problems were obviously higher than internalizing behavior problems before adenotonsillectomy. Adenotonsillectomy in this patient did not result in significant changes in externalizing and internalizing behavior problems and all subscores of CBCL became below the cutoff point of clinical level, 70. In this regard, Lewin et al. [39] reported that externalizing behavior problems in CBCL were common in patients with mild OSAS. Our results are compatible with the previous findings. Longitudinal studies of large sample size, starting at young age, are needed in order to explore the long-term consequences of OSAS in children and determine the specific effects of pediatric OSAS on neurocognitive functions.

The present study has several limitations. As we reevaluated neurocognitive functions 4 months after the treatment, practice effect with regard to the neurocognitive function tests could have influenced the results. The interval between the tests before and after the treatment may have been short to confirm the effectiveness of adenotonsillectomy. A randomized trial of adenotonsillectomy for childhood sleep apnea reported that after a 7-month intervention period, school-age children with prolonged oxyhemoglobin desaturation, who underwent surgery, did not show the significant improvement in attention or executive function measured by neuropsychological testing, although the improvements of behavior, quality of life, and polysomnographic findings were observed [40]. In the meta-analysis, the duration between assessment before and after adenotonsillectomy was wide range from 2.4 months to 18 months [8]. The optimal interval for assessment before and after surgery was necessary to be determined. According to Chervin et al, there could be persistent OSA at 1 year follow-up in up to 20% of patients with OSAS [41]. The AHI of 1.8 in our study might be a residual OSAS. Longitudinal follow-up is necessary to determine when recovery is maximal. More cases need to be examined to confirm the findings of the present study.

## 4. Conclusion

We reported a child initially suspected of having ADHD with drug resistant enuresis. Treatment of OSAS with adenotonsillectomy significantly improved snoring, enuresis, daytime behavioral problems, and neurocognitive functions. In order to identify pediatric OSAS, underlying neurodevelopmental disorders like ADHD, medical interview for snoring, and the oropharyngeal examination are important.

## Figures and Tables

**Table 1:** Polysomnographic data before and after treatment.

	Pretreatment	Posttreatment
Polysomnography		
Total recording time (min)	537	497
Total sleep time (min)	494	477
Sleep time (min)	512	490
Sleep efficiency (%)	89.6	96.4
Sleep latency (min)	27	4
REM latency (min)	91	110
Arousal (/h)	3.6	2.8
Stage I NREM sleep (%)	8.6	6.9
Stage II NREM sleep (%)	42.9	39.2
Stage III/IV NREM sleep (%)	23.7	24.0
REM sleep (%)	21.2	27.5
Apnea/hypopnea index	21.9	1.8
Multiple sleep latency test		
Sleep latency time (min)		
1st nap	20.0	13.5
2nd nap	18.0	20.0
3rd nap	5.0	12.5
4th nap	20.0	20.0
5th nap	8.0	20.0
Mean sleep latency	14.2	17.2
Sleep-onset REM period	—	—

**Table 2:** Kaufman Assessment Battery for children (K-ABC) before and after treatment.

	Pretreatment	Posttreatment
Sequential Processing Scale	123	125
Simultaneous Processing Scale	95	112
Mental Processing Composite Scale	109	122
Achievement Scale	125	124
Nonverbal Scale	93	100

**Table 3:** Rey-Osterrieth Complex Figure Test (ROCF) and Rey's Auditory Verbal Learning Test (RAVLT) before and after treatment.

	Pretreatment	Posttreatment
ROCF (/36 score)		
Copy	33.0	35.0
Immediate recall	11.5	20.5
Delayed recall	11.5	20.5
RAVLT	6-6-11-12-13-(4)-11	7-12-15-15-15-(7)-15
Recognition	15/15	15/15
Errors	0	0

ROCF involves copying complex geometric figures and then reproducing them from memory, immediately and after a brief delay. It is a count of the number of parts reproduced irrespective of organizational integrity.

RAVLT involves listening to 15 words and recalling them from memory. This performance is repeated 7 times, and on the 6th trial another set of 15 words is used and the total number of correctly recalled words is scored.
